# 312 nm UVB Phototherapy Limits Atherosclerosis by Regulating Immunoinflammatory Responses in Mice

**DOI:** 10.24546/0100492952

**Published:** 2025-02-03

**Authors:** AGA KRISNANDA, NAOTO SASAKI, KEN ITO, TORU TANAKA, MASAKAZU SHINOHARA, HILMAN ZULKIFLI AMIN, SAYO HORIBE, MOTOAKI IWAYA, KEN-ICHI HIRATA, ATSUSHI FUKUNAGA, YOSHIYUKI RIKITAKE

**Affiliations:** 1 Laboratory of Medical Pharmaceutics, Kobe Pharmaceutical University, Kobe, Japan; 2 Division of Cardiovascular Medicine, Department of Internal Medicine, Kobe University Graduate School of Medicine, Kobe, Japan; 3 Division of Molecular Epidemiology, Kobe University Graduate School of Medicine, Kobe, Japan; 4 The Integrated Center for Mass Spectrometry, Kobe University Graduate School of Medicine, Kobe, Japan; 5 Department of Cardiovascular Medicine, National Cerebral and Cardiovascular Center, Suita, Osaka, Japan; 6 Faculty of Medicine, Universitas Indonesia, Jakarta, Indonesia; 7 Department of Materials Science and Engineering, Meijo University, Nagoya, Japan; 8 Department of Cardiology, Kakogawa Central City Hospital, Kakogawa, Hyogo, Japan; 9 Department of Dermatology, Division of Medicine for Function and Morphology of Sensory Organs, Faculty of Medicine, Osaka Medical and Pharmaceutical University, Takatsuki, Osaka, Japan

**Keywords:** Atherosclerosis, Ultraviolet B, Immunology, T cells, Inflammation

## Abstract

**AIM:**

Our previous studies identified ultraviolet B (UVB) irradiation as a possible approach for preventing atherosclerosis. The aim of this study was to clarify the effect of 312 nm UVB, a wavelength similar to that of clinically available narrow-band UVB for the treatment of psoriasis, on atherosclerosis and the underlying mechanisms.

**METHODS AND RESULTS:**

Using a recently developed UVB-light-emitting diode device, we irradiated 6-week-old male atherosclerosis-prone apolipoprotein E-deficient mice with 312 nm UVB at 5 or 10 kJ/m^2^ and examined its effect on the development of atherosclerosis and immunoinflammatory responses by performing histological analysis, flow cytometry, biochemical assays, and liquid chromatography/mass spectrometry-based lipidomics. UVB irradiation at 10 kJ/m^2^ but not at 5 kJ/m^2^ significantly attenuated the development of aortic root atherosclerotic plaques, while UVB irradiation at both doses induced a less inflammatory plaque phenotype. This atheroprotective effect was associated with a reduced effector T cell number, a shift toward anti-atherogenic helper T cell responses, and increased proportion of regulatory T cells in lymphoid tissues and increased levels of proresolving lipid mediators in the skin.

**CONCLUSIONS:**

We demonstrated that 312 nm UVB irradiation limits atherosclerosis by favorably modulating the T cell balance and lipid mediator profile. Our findings indicate that 312 nm UVB phototherapy could be an attractive immunomodulatory approach for preventing and treating atherosclerosis.

## INTRODUCTION

Atherosclerosis is responsible for fatal diseases such as coronary artery disease (CAD) and stroke which are major causes of mortality in developed and developing countries. Even though patients at high risk of these diseases receive advanced medical treatment, they still have substantial residual risk that could be derived from arterial immunoinflammatory responses (1). Emerging clinical evidence suggests the efficacy of anti-inflammatory treatment for the prevention of CAD in patients who previously suffered from this disease (2). Critical involvement of immunoinflammatory responses in the arteries, caused by dysregulated innate and adaptive immunity, in the development of atherosclerosis is no longer controversial (3). Clinical research on carotid artery atherosclerotic plaques has identified activated and differentiated T cells as the major components of the accumulated immune cells, which indicates a possible role for effector T cell (Teff) immune responses in the development of human atherosclerotic plaques (4). The data from experimental studies also suggest that Teff immune responses are deeply involved in the pathogenesis of atherosclerosis. Among helper T cell subsets such as T helper type 1 (Th1), T helper type 2 (Th2), and T helper type 17 (Th17) cells, Th1 cells reportedly promote atherosclerosis by secreting proinflammatory interferon (IFN)-γ (3). Although the role of Th2-mediated immune responses remains still controversial, a shift in the immune balance of Th1 cells and Th2 cells toward Th1 cell responses is thought to contribute to the development of atherosclerosis (5). On the other hand, solid experimental evidence indicates that regulatory T cells (Tregs), defined by the expression of the transcription factor forkhead box P3 (Foxp3), play an anti-atherogenic role by controlling immunoinflammatory responses or improving lipid metabolism (6). Accumulating clinical evidence indicates the possible involvement of Tregs in the protection against CAD (6, 7). Previous experimental studies have attempted to modulate the Teff/Treg balance via therapies involving antibodies (8–10), cytokines (10), and an active form of vitamin D_3_ (11), or vaccination strategies (12). They have demonstrated that shifting the Teff/Treg balance toward Treg responses could be a possible approach for limiting the progression of atherosclerosis. However, therapeutic strategies aimed at modulating the T cell balance have not been established in clinical practice due to the potential risk of immune dysregulation.

In daily life, we receive sunlight including ultraviolet B (UVB), which is indispensable for the maintenance of human health. As UVB irradiation plays an essential role in vitamin D synthesis in the skin and in the regulation of the immune system, it has received much attention. Notably, UVB-based phototherapy is a clinically effective treatment for immunoinflammatory cutaneous diseases including psoriasis (13). We previously reported that irradiation with either broad-band UVB (a continuous spectrum from 280 to 320 nm with a peak at approximately 313 nm) (14) or 282 nm wavelength of UVB (a narrow peak at approximately 282) (15) augments anti-inflammatory immune responses including Treg responses and reduces atherosclerotic plaque development in hypercholesterolemic apolipoprotein E-deficient (*Apoe^−/−^*) mice. Narrow-band UVB (a narrow peak at approximately 311 nm) therapy is clinically effective for the treatment of psoriasis and is considered as a preferred alternative to broad-band UVB therapy (13). Notably, our recent study showed that irradiation with 2 kJ/m^2^ 312 nm UVB (a narrow peak at approximately 312 nm), a wavelength similar to that of clinically used narrow-band UVB, tended to attenuate atherosclerotic lesion formation, although this effect did not reach a statistical significance and was not associated with Treg responses (15). Given that the UVB dose used in this previous work was relatively low compared with that used for clinical treatment, we are interested in the effect of a higher dose of 312 nm UVB irradiation on immunoinflammatory responses and atherosclerosis.

This study aimed to investigate the effect of 312 nm UVB irradiation on the development of atherosclerosis and the underlying mechanisms in hypercholesterolemic mice, with a particular focus on immunoinflammatory responses including T cell immune responses and lipid mediator production.

## MATERIALS AND METHODS

### Animals

All mice were male on a C57BL/6 background and fed a standard chow diet (CLEA, Tokyo, Japan). *Apoe^−/−^* mice are previously described (8). We housed mice in a cage for each treatment group in a specific pathogen-free animal facility at Kobe Pharmaceutical University. All animal experiments were approved and registered by the Animal Care Committee of Kobe Pharmaceutical University (Permit Numbers: 2021-034, 2022-006, 2023-036, and 2024-012) and conformed to the National Institutes of Health Guide for the Care and Use of Laboratory Animals and the ARRIVE guidelines (Animal Research: Reporting of *In Vivo* Experiments).

### Methods

#### UVB irradiation

A light-emitting diode (LED) lamp (Nikkiso Co., Ltd., Japan) that can emit 312 nm a wavelength of UVB was previously developed (15) and used in this study. The irradiance of the 312 nm UVB was 2.52 J/m^2^/second at a distance of 14 cm. The mice were placed 14 cm below the bank of lamps and were irradiated for the indicated weeks after shaving their backs in the animal facility, while nonirradiated mice were not subjected to these procedures. The UVB irradiation dose used in this study was 5 or 10 kJ/m^2^ as indicated. Randomization and allocation concealment were performed. Littermate mice were equally allocated to each treatment group. During the experiments, animal/cage location was not controlled. Investigators were not blinded to treatment allocation. Criterion for exclusion was defined as serious burns, but during at least 3 observations per week we did not observe such symptoms, and no UVB-irradiated mice were excluded. UVB irradiation was performed in our animal facility and other experimental procedures were performed in our laboratory rooms.

#### Assessment of biochemical parameters

The plasma lipid profile was determined as described previously (15). The plasma C-reactive protein levels were measured using a Mouse C-Reactive Protein/CRP Quantikine ELISA Kit according to the manufacturer’s instructions (R&D Systems).

#### Assessment of atherosclerotic lesions

To examine the effect of specific wavelengths of UVB on atherosclerosis, we irradiated 6-week-old male *Apoe^−/−^* mice with 312 nm UVB once weekly for 14 weeks and analyzed atherosclerotic lesions in the aortic root and thoracoabdominal aorta at 20 weeks of age. Mice were anesthetized by intraperitoneal injection of medetomidine hydrochloride (0.3 mg/kg), midazolam (4 mg/kg), and butorphanol tartrate (5 mg/kg) (all from WAKO, Osaka, Japan), and the aorta was perfused with saline. The aorta was dissected from the middle of the left ventricle to the bifurcation of the iliac artery. For aortic root lesion analysis, samples were cut from the ascending aorta, and proximal samples containing the aortic sinus were collected. Atherosclerotic lesions of the aortic sinus and thoracoabdominal aortas were analyzed as described previously (15).

#### Histological analysis of atherosclerotic lesions

Immunohistochemistry was performed on 4% paraformaldehyde-fixed cryosections (10 μm) of mouse aortic roots using antibodies to identify macrophages (MOMA-2, 1:400; BMA Biomedicals) and T cells (CD4, 1:100; BD Biosciences), followed by detection with biotinylated secondary antibodies and streptavidin-horseradish peroxidase. Staining with Masson’s trichrome was used to delineate the fibrous area. The stained sections were analyzed as described previously (15).

#### Flow cytometry

For flow cytometric analysis of lymphoid tissues, skin-draining lymph node (LN) cells and splenocytes were isolated and stained in PBS containing 2% fetal calf serum as described previously (15). We used axillary and inguinal LNs as skin-draining LNs. Intracellular staining of Foxp3 was performed using a Foxp3 staining buffer set (Thermo Fisher Scientific) according to the manufacturer’s instructions. In some experiments, splenocytes were stimulated with 20 ng/ml phorbol 12-myristate 13-acetate (Sigma) and 1 mmol/L ionomycin (Sigma) for 5 hours in the presence of Brefeldin A (Thermo Fisher Scientific) and intracellular cytokine staining was performed as described previously (15). Flow cytometric analysis was performed by FACSAria III (BD Biosciences) using FlowJo software version 10.10.0 (Tree Star). The antibodies used are listed in Table I.

#### Liquid chromatography/mass spectrometry/mass spectrometry-based lipidomics

Various lipid mediators in the skin were analyzed by liquid chromatography/mass spectrometry (MS)/MS-based lipidomics as described previously (16).

#### Statistical analysis

Normality was assessed by Shapiro–Wilk normality test. Mann–Whitney *U*-test or 2-tailed Student’s or Welch’s *t*-test was used to detect significant differences between 2 groups where appropriate. One-way ANOVA followed by Dunnett’s post hoc test or 2-way ANOVA followed by Dunnett’s post hoc test was performed for multiple groups where appropriate. A value of *P* < 0.05 was considered statistically significant. Data were expressed as means ± s.d. No data were excluded from the analysis. Investigators were not blinded to the data analysis. For statistical analysis, GraphPad Prism version 9.0 (GraphPad Software Inc.) was used.

## RESULTS

### 312 nm UVB irradiation inhibits the development of atherosclerosis and induces a less inflammatory plaque phenotype

**Fig. 1:**
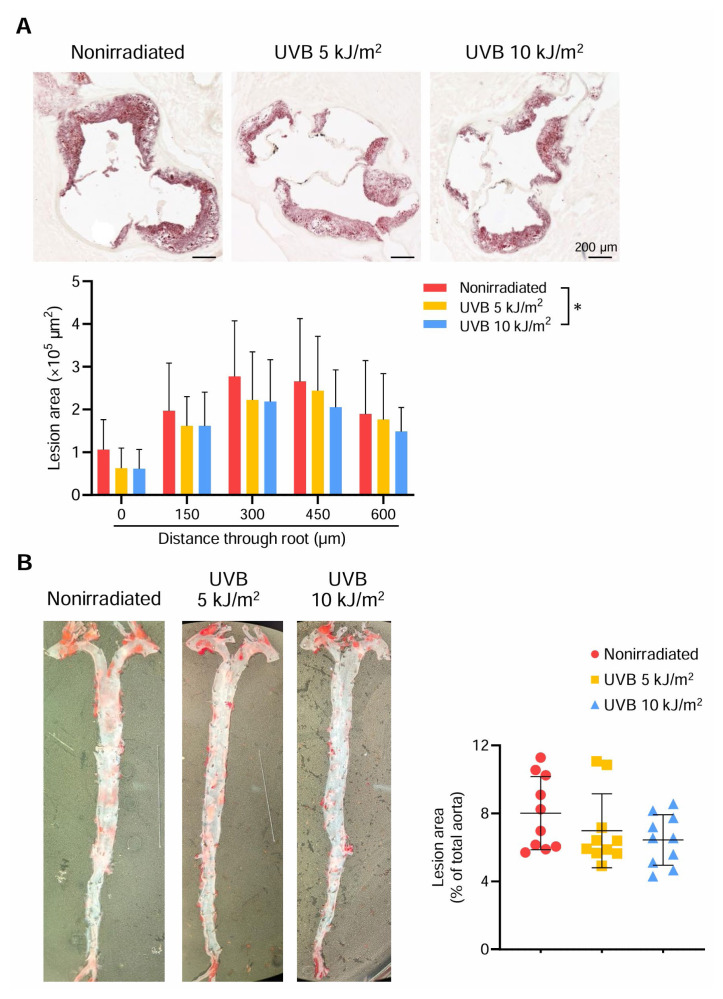
312 nm ultraviolet B (UVB) irradiation inhibits the development of atherosclerosis Six-week-old male apolipoprotein E-deficient (*Apoe*^−/−^) mice were irradiated with 312 nm UVB at 5 or 10 kJ/m^2^ once weekly for 14 weeks and euthanized at 20 weeks of age, and atherosclerotic lesions were assessed. Nonirradiated male *Apoe*^−/−^ mice served as controls. **A**, Representative photomicrographs of Oil Red O staining and quantitative analysis of atherosclerotic lesion area at 5 different levels in the aortic sinus of 312 nm UVB-irradiated or nonirradiated mice. n = 11 per group. **B**, Representative photomicrographs of Oil Red O staining and quantitative analysis of atherosclerotic lesion area in the thoracoabdominal aorta of 312 nm UVB-irradiated or nonirradiated mice. n = 10 per group. Black bars represent 200 μm. Data points represent individual animals. Horizontal bars represent means. Error bars indicate s.d. Data are expressed as the mean ± s.d. (**A**). *P < 0.05; 2-way ANOVA followed by Dunnett’s post hoc test.

During the course of experiment, we did not observe any adverse effects including skin cancer or burns. No significant differences in body weight, plasma lipid profile, and plasma levels of C-reactive protein were found between 5 or 10 kJ/m^2^ 312 nm UVB-irradiated and nonirradiated mice (Table II). Notably, compared with nonirradiated mice, 10 kJ/m^2^ 312 nm UVB-irradiated mice exhibited a significant reduction in atherosclerotic lesion size in the aortic sinus when the aortic sinus plaques at 5 different levels were analyzed in detail (Fig. 1A). On the other hand, irradiation with the lower 5 kJ/m^2^ 312 nm UVB did not significantly affect atherosclerosis (Fig. 1A). Means ± s.d. of the mean plaque area at 5 different levels was 2.07 ± 0.69 × 10^5^ μm^2^ in control nonirradiated mice, 1.73 ± 0.70 × 10^5^ μm^2^ in 5 kJ/m^2^ UVB-irradiated mice, and 1.59 ± 0.62 × 10^5^ μm^2^ in 10 kJ/m^2^ UVB-irradiated mice.

In parallel with the cross-sectional experiments, we conducted en face analysis of thoracoabdominal aortas, which revealed no difference in the aortic plaque burden between 5 or 10 kJ/m^2^ UVB-irradiated and nonirradiated mice (Fig. 1B). Means ± s.d. of the mean atherosclerotic lesion area in the aorta was 8.02 ± 2.15% in control nonirradiated mice, 6.98 ± 2.17% in 5 kJ/m^2^ UVB-irradiated mice, and 6.44 ± 1.49% in 10 kJ/m^2^ UVB-irradiated mice, as shown in Fig. 1B.

**Fig. 2:**
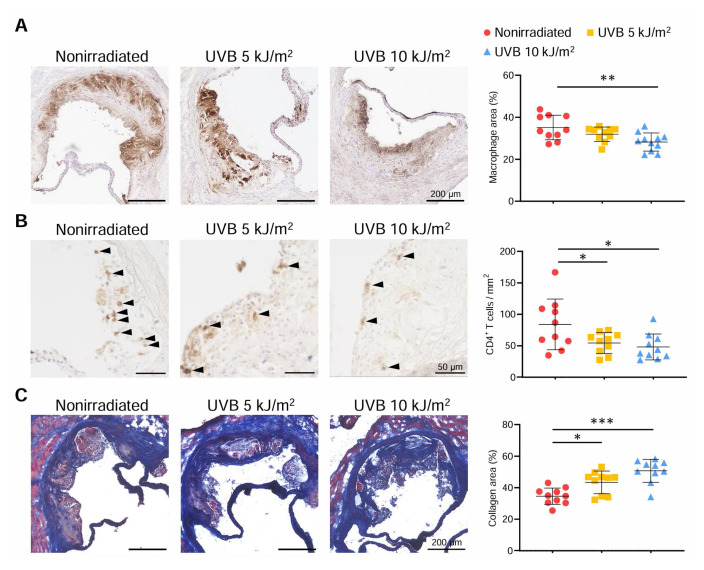
312 nm ultraviolet B (UVB) irradiation induces a less inflammatory plaque phenotype Six-week-old male apolipoprotein E-deficient (*Apoe*^−/−^) mice were irradiated with 312 nm UVB at 5 or 10 kJ/m^2^ once weekly for 14 weeks and euthanized at 20 weeks of age, and atherosclerotic lesions were assessed. Nonirradiated male *Apoe*^−/−^ mice served as controls. **A**–**C**, Representative sections and quantitative analyses of MOMA-2^+^ macrophages (**A**), CD4^+^ T cells (**B**), and collagen (**C**) in the aortic sinus. Black arrowheads indicate the CD4^+^ T cells. n = 10 per group. Black bars represent 50 or 200 μm as described. Data points represent individual animals. Horizontal bars represent means. Error bars indicate s.d. *P < 0.05; **P < 0.01; ***P < 0.001; 1-way ANOVA followed by Dunnett’s post hoc test.

To determine the effect of 312 nm UVB on atherosclerotic plaque components, we performed immunohistochemical studies of atherosclerotic lesions in the aortic sinus. Compared with those of nonirradiated mice, the atherosclerotic lesions of 10 kJ/m^2^ UVB-irradiated mice showed markedly reduced accumulation of macrophages by 20% and of CD4^+^ T cells by 43% (Fig. 2A and 2B). Although intraplaque macrophage accumulation did not change, CD4^+^ T cell infiltration was significantly lower in 5 kJ/m^2^ UVB-irradiated mice than in nonirradiated mice (Fig. 2A and 2B). To evaluate collagen fibers in the aortic sinus plaques, we performed Masson’s trichrome staining. The proportion of collagen content was significantly higher in either 5 or 10 kJ/m^2^ UVB-irradiated mice than in nonirradiated mice (Fig. 2C).

These results collectively suggest that although the atheroprotective effect seems to be limited to aortic sinus lesions, the higher 10 kJ/m^2^ 312 nm UVB irradiation inhibits the development of atherosclerosis possibly through a reduction in proinflammatory immune responses in the aorta.

### 312 nm UVB irradiation shifts the Teff/Treg balance toward Treg responses in peripheral lymphoid tissues

**Fig. 3:**
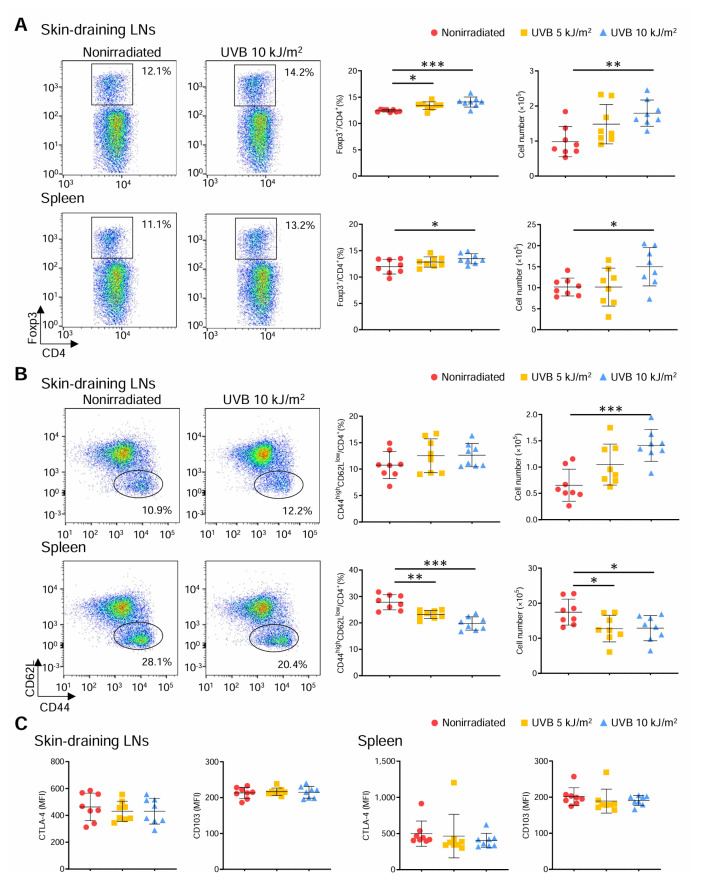
312 nm ultraviolet B (UVB) irradiation shifts the effect T cell/regulatory T cell (Treg) balance toward Treg responses in peripheral lymphoid tissues Male apolipoprotein E-deficient (*Apoe*^−/−^) mice were irradiated with 312 nm UVB at 5 or 10 kJ/m^2^ once weekly for 6 weeks. Nonirradiated male *Apoe*^−/−^ mice served as controls. Four days after the last UVB irradiation, lymphoid cells from skin-draining lymph nodes (LNs) and spleen were prepared. **A** and **B**, Representative flow cytometric analysis of CD4^+^ forkhead box P3 (Foxp3)^+^ Tregs (**A**) and CD4^+^CD44^high^CD62L^low^ effector memory T cells (**B**) in the skin-draining LNs and spleen. The graphs represent the proportions and total numbers of CD4^+^Foxp3^+^ Tregs (**A**) and CD4^+^CD44^high^CD62L^low^ effector memory T cells (**B**) in the skin-draining LNs and spleen. n = 8 per group. **C** and **D**, The expression levels of cytotoxic T lymphocyte-associated antigen-4 (CTLA-4) and CD103 were analyzed by gating on CD4^+^Foxp3^+^ Tregs in the skin-draining LNs (**C**) and spleen (**D**). n = 8 per group. Data points represent individual animals. Horizontal bars represent means. Error bars indicate s.d. *P < 0.05; **P < 0.01; ***P < 0.001; 1-way ANOVA followed by Dunnett’s post hoc test. MFI indicates mean fluorescence intensity.

To elucidate the atheroprotective effect of 10 kJ/m^2^ 312 nm UVB irradiation, we first focused on investigating CD4^+^ T cell responses in peripheral lymphoid tissues. We irradiated male *Apoe^−/−^* mice with 5 or 10 kJ/m^2^ 312 nm UVB once weekly for 6 weeks and examined CD4^+^Foxp3^+^ Treg and CD4^+^CD44^high^CD62L^low^ effector memory T cell populations in the skin-draining LNs and spleen by flow cytometry. Notably, 10 kJ/m^2^ UVB irradiation significantly increased the frequency and number of CD4^+^Foxp3^+^ Tregs in the skin-draining LNs of *Apoe^−/−^* mice, while 5 kJ/m^2^ UVB irradiation increased only the frequency of CD4^+^Foxp3^+^ Tregs in the skin-draining LNs (Fig. 3A). In addition, the frequency and number of CD4^+^Foxp3^+^ Tregs were also increased in the spleen of 10 kJ/m^2^ UVB-irradiated mice, while 5 kJ/m^2^ UVB irradiation had no effect (Fig. 3A).

Interestingly, the frequency and number of CD4^+^CD44^high^CD62L^low^ effector memory T cells in the spleen of 10 kJ/m^2^ UVB-irradiated mice were significantly lower than those in the spleen of nonirradiated mice, although the number of CD4^+^CD44^high^CD62L^low^ effector memory T cells in the skin-draining LNs was higher in 10 kJ/m^2^ UVB-irradiated mice (Fig. 3B). Similar findings were obtained for the frequency and number in the spleen of 5 kJ/m^2^ UVB-irradiated mice (Fig. 3B).

We further investigated the effect of 312 nm UVB on the functionality and activation state of CD4^+^Foxp3^+^ Tregs. We analyzed the expression of suppressive function-associated molecule cytotoxic T lymphocyte-associated antigen-4 (CTLA-4) and activation-associated molecule CD103 in CD4^+^Foxp3^+^ Tregs in peripheral lymphoid tissues by flow cytometry. However, there were no significant changes in the expression of either molecule in CD4^+^Foxp3^+^ Tregs in the skin-draining LNs and spleen of 5 or 10 kJ/m^2^ UVB-irradiated mice (Fig. 3C), implying a minor effect on the suppressive capacity of CD4^+^Foxp3^+^ Tregs.

Taken together, these data suggest that the higher 10 kJ/m^2^ UVB irradiation efficiently shifts the Teff/Treg balance toward Treg responses by systemically reducing Teff frequency and number and increasing Treg frequency and number, which could contribute to the reduction of atherosclerosis. Although the lower 5 kJ/m^2^ UVB irradiation resulted in a systemic decrease in Teffs, it had a minor effect on systemic Treg responses, implying a modest change in the Teff/Treg balance and weak atheroprotective effects.

### 312 nm UVB irradiation decreases the Th1/Th2 ratio without affecting other immune cell responses in peripheral lymphoid tissues

**Fig. 4:**
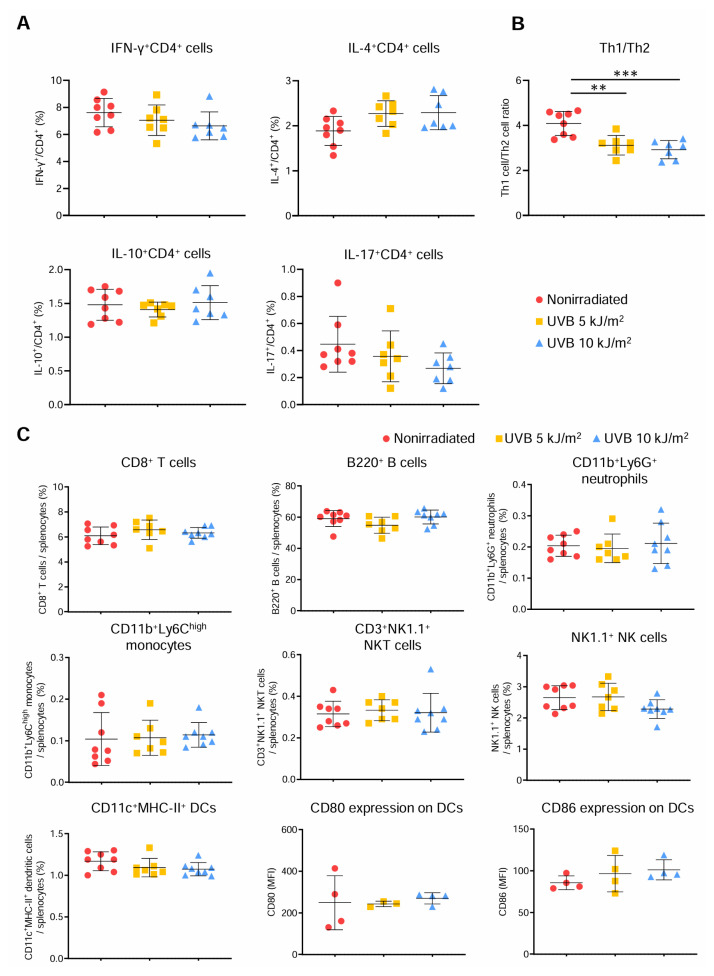
312 nm ultraviolet B (UVB) irradiation decreases the ratio of T helper type 1 (Th1) to T helper type 2 (Th2) without affecting other immune cell responses in peripheral lymphoid tissues Male apolipoprotein E-deficient (*Apoe*^−/−^) mice were irradiated with 312 nm UVB at 5 or 10 kJ/m^2^ once weekly for 6 weeks. Nonirradiated male *Apoe*^−/−^ mice served as controls. Four days after the last UVB irradiation, lymphoid cells from spleen were prepared. **A** and **B**, Lymphoid cells from spleen were stimulated with phorbol 12-myristate 13-acetate and ionomycin *in vitro*. Intracellular cytokine staining was performed. The graphs represent the frequencies of interferon (IFN)-γ^+^, interleukin (IL)-4^+^, IL-10^+^, and IL-17^+^ CD4^+^ T cells (**A**) and the ratio of Th1 cells to Th2 cells (**B**) in spleen. n = 7 to 8 per group. **C**, Proportions of splenic CD8^+^ T cells, B220^+^ B cells, CD11b^+^Ly6G^+^ neutrophils, CD11b^+^Ly6C^high^ monocytes, natural killer (NK) T cells, NK cells, and CD11c^+^ major histocompatibility complex (MHC)-II^+^ dendritic cells (DCs), and the expression of CD80 and CD86 on CD11c^+^MHC-II^+^ DCs were determined by flow cytometry. n = 7 to 8 per group for immune cell proportions and 4 per group for CD80/86 expression. Data points represent individual animals. Horizontal bars represent means. Error bars indicate s.d. **P < 0.01; ***P < 0.001; 1-way ANOVA followed by Dunnett’s post hoc test. MFI indicates mean fluorescence intensity.

Since we found that 312 nm UVB irradiation modulated Treg and Teff populations, we next sought to determine whether it also affects CD4^+^ T cell subsets. We irradiated male *Apoe*^−/−^ mice with 5 or 10 kJ/m^2^ 312 nm UVB once weekly for 6 weeks and analyzed CD4^+^ T cell subsets in spleen by intracellular cytokine staining. The frequencies of IFN-γ-producing Th1 cells, IL-4-producing Th2 cells, IL-10-producing CD4^+^ T cells, and IL-17-producing Th17 cells in spleen were not different between 5 or 10 kJ/m^2^ UVB-irradiated and nonirradiated mice (Fig. 4A). Interestingly, the ratio of IFN-γ-producing Th1 cells to IL-4-producing Th2 cells (Th1/Th2 ratio) was significantly decreased in both 5 and 10 kJ/m^2^ 312 nm UVB-irradiated mice, although there were no differences in the proportions of the individual subsets (Fig. 4A and 4B). We also examined the proportions of other immune cells and the expression of CD80 and CD86 on dendritic cells in spleen by flow cytometry, and found that 5 or 10 kJ/m^2^ UVB irradiation had no significant effects on other immune cell responses (Fig. 4C).

These results suggest that the immunomodulatory effect of 312 nm UVB irradiation is favorable for modulating the balance of T cells including Tregs and helper T cells, and may be specific to T cell responses, indicating that 312 nm UVB irradiation could avoid unwanted immunosuppression caused by general dysregulation of innate or adaptive immune responses.

### 312 nm UVB irradiation increases levels of proresolving lipid mediators in the skin

**Fig. 5:**
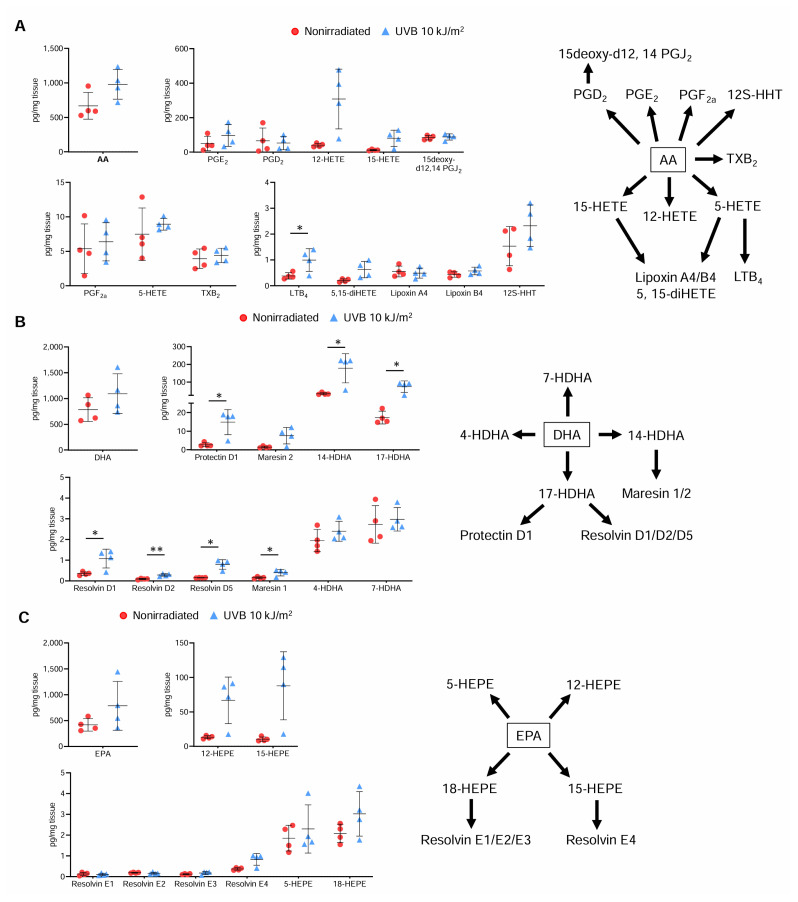
312 nm ultraviolet B (UVB) irradiation increases levels of proresolving lipid mediators in the skin Male apolipoprotein E-deficient (*Apoe*^−/−^) mice were irradiated with 312 nm UVB at 10 kJ/m^2^ once weekly for 6 weeks. Nonirradiated male *Apoe*^−/−^ mice served as controls. Four days after the last UVB irradiation, the back skin of the mice was isolated. The production of various lipid mediators in the skin was analyzed by liquid chromatography/mass spectrometry/mass spectrometry-based lipidomics. **A**–**C**, Quantification of arachidonic acid (AA) and its derived lipid mediators (**A**), docosahexaenoic acid (DHA) and its derived lipid mediators (**B**), and eicosapentaenoic acid (EPA) and its derived lipid mediators (**C**) in the skin. Schematics show metabolic pathways related to AA (**A**), DHA (**B**), and EPA (**C**). n = 4 per group. Data points represent individual animals. Horizontal bars represent means. Error bars indicate s.d. *P < 0.05; Mann–Whitney *U*-test: protectin D1, 14-HDHA, and 17-HDHA (**B**); 2-tailed Student’s *t*-test: LTB_4_ (**A**) and resolvin D2 and maresin 1 (**B**); 2-tailed Welch’s *t*-test: resolvin D1 and resolvin D5 (**B**). LTB_4_ indicates leukotriene B_4_; PGD_2_, prostaglandin D_2_; PGE_2_, prostaglandin E_2_; PGF_2a_, prostaglandin F_2a_; and TXB_2_, thromboxane B_2_.

Accumulating evidence has linked the imbalance between proresolving and proinflammatory lipid mediators to the progression of chronic inflammatory diseases including atherosclerosis (17). We found that 312 nm UVB irradiation had a modest effect on the amount of arachidonic acid and its derived lipid mediators in the skin (Fig. 5A). Interestingly, the amount of docosahexaenoic acid in the skin did not change, while the amount of its derived proresolving lipid mediators including resolvin D1, resolvin D2, resolvin D5, maresin 1, and protectin D1 was increased by UVB irradiation (Fig. 5B). The amount of eicosapentaenoic acid was not affected by UVB irradiation, while the amount of its derived proresolving resolvin E4 tended to increase in UVB-irradiated mice (Fig. 5C). These results indicate that increased amounts of proresolving lipid mediators in the skin may contribute to attenuating atherosclerotic lesion formation.

## DISCUSSION

Accumulating experimental and clinical evidence suggests that anti-inflammatory treatment could be a possible approach for preventing CAD (1). However, clinical therapies directly targeting immunoinflammatory responses have not been established due to adverse effects such as infection and the inability to prevent fatal cardiac events. In this study, using our unique UVB-LED device which resembles clinically established narrow-band UVB therapy, we demonstrated that 312 nm UVB irradiation attenuated the development of aortic root atherosclerotic plaques and increased plaque stability in hypercholesterolemic mice by effectively shifting the Teff/Treg balance toward anti-inflammatory Treg responses and improving the atherosclerosis-related Th1/Th2 balance. This atherosclerosis reduction was also associated with the augmented production of proresolving lipid mediators in the skin. Given that a phototherapy with a wavelength similar to that of 312 nm UVB has already been established as an effective clinical approach for treating various cutaneous diseases, the newly developed 312 nm UVB therapy could be an attractive strategy for treating and preventing CAD.

Experimental and clinical data indicate that pathogenic immune responses mediated by Teffs including IFN-γ-producing Th1 cells critically accelerate vascular inflammation and atherosclerosis (3). A skewed immune balance of Th1 and Th2 cells toward Th1 cell responses is thought to exacerbate atherosclerosis (5). By contrast, Foxp3-expressing Tregs play an anti-atherogenic role by regulating immunoinflammatory responses derived from pathogenic innate and adaptive immunity (6). In this study, we found that 10 kJ/m^2^ 312 nm UVB irradiation increased the proportion and number of Tregs and decreased the proportion and number of Teffs and the Th1/Th2 ratio in lymphoid tissues of hypercholesterolemic mice, which resulted in reduced inflammation and atherosclerotic plaque development in the aortic root. Similar findings were obtained for Teffs and the Th1/Th2 ratio in the lower 5 kJ/m^2^ 312 nm UVB-irradiated mice, while there were minor effects on Tregs and atherosclerosis in these mice, which is consistent with the findings of our recent study using the same UVB irradiation at a lower dose of 2 kJ/m^2^ (15). Given that Tregs fail to control Teff responses under inflammatory conditions (18), it is likely that a combination approach involving the downregulation of Teff responses and the augmentation of Treg responses can effectively limit atherosclerosis. This idea is supported by our previous study in hypercholesterolemic *Apoe*^−/−^ mice showing that administration of anti-CD3 antibodies and IL-2 complexes dramatically expanded Tregs by reducing Teff responses and potently attenuated the development of atherosclerosis (10). These results collectively indicate that the synergistic effects of augmented Treg responses and downregulated Teff responses may be critical for the reduction in atherosclerosis induced by our 10 kJ/m^2^ 312 nm UVB therapy. Considering that the UVB doses used in this study are within the range of clinical use for the treatment of skin disease, 312 nm UVB phototherapy may be safely applied for the treatment of atherosclerotic disease. We recently reported that 282 nm UVB irradiation at 2 kJ/m^2^ augmented Treg responses and reduced atherosclerotic plaque development but had no significant effect on Teff responses in hypercholesterolemic mice (15). Accordingly, we suppose that the effect of UVB irradiation on T cell immunity and atherosclerosis may differ depending on the wavelength or dose.

Based on its diverse immune-modulating effects (19), UVB-based phototherapy is a clinically established treatment for various cutaneous diseases and has few severe adverse effects if the irradiation conditions are carefully controlled. We recently revealed the anti-atherogenic effects of 282 nm UVB irradiation. This indicates that the therapeutic efficacy of UVB irradiation for atherosclerotic disease and other cutaneous inflammatory diseases may differ depending on its wavelength. Our finding of the novel anti-atherogenic action of 282 nm UVB, at first glance, appears to be interesting because this shorter 282 nm UVB does not reach the ground. However, the detailed biological actions and safety of 282 nm UVB irradiation remain to be determined and further clarification is needed. Considering the clinical efficacy and few serious adverse effects of the narrow-band UVB therapy (13), 312 nm UVB phototherapy may be applied for the treatment of atherosclerotic disease. Although how narrow-band UVB irradiation controls the cutaneous disease state is not completely known, a previous clinical study in patients with psoriasis reported that phototherapy with UVA or narrow-band UVB significantly improved the disease state in association with the expansion of peripheral Tregs (20). Our study supports previous clinical findings and may provide additional detailed mechanisms for the therapeutic effect of narrow-band UVB irradiation on cutaneous diseases, even though our results were obtained using an atherosclerotic mouse model. Importantly, psoriasis patients have an increased risk of CAD, which may be related to chronic inflammation including pathogenic Th1 or Th17 immune responses (21). Our data suggest that 312 nm or narrow-band UVB phototherapy may mitigate inflammation not only in the skin but also systemically. In this study, we irradiated the entire backs of mice with 312 nm UVB, whereas the irradiation area was limited to the inflammatory lesions on the skin in psoriasis patients. This may indicate a lesser effect of UVB therapy on T cell immune responses and atherosclerosis in clinical situations, although it is unclear whether the irradiation area size is critical for exerting the anti-atherogenic effects. It will be intriguing to expand the irradiation area in these patients and investigate the preventive effect on CAD in future clinical trials.

Conventional anti-inflammatory therapies including statins are known to reduce C-reactive protein levels and atherosclerotic cardiovascular disease events by inhibiting proinflammatory cytokine production. On the other hand, we found no significant difference in plasma C-reactive protein levels between 5 or 10 kJ/m^2^ 312 nm UVB-irradiated and nonirradiated mice, indicating that 312 nm UVB treatment may exert its anti-atherogenic effects through different mechanisms. Given our finding of the dominant effect on T cell responses, we think that the main mechanism for the anti-atherogenic action of 312 nm UVB phototherapy may be favorable modulation of the T cell balance between proinflammatory T cells and anti-inflammatory Tregs, which leads to reduced inflammation through limiting excessive immunoinflammatory responses.

Proresolving lipid mediators are known to be important plaque-stabilizing factors. Interestingly, we found a significant increase in the levels of various proresolving lipid mediators in the skin of 312 nm UVB-irradiated mice, while a modest change in the levels of proinflammatory lipid mediators was observed. It has been reported that proresolving lipid mediators such as resolvin D1, resolvin D2, and maresin 1, whose levels are markedly increased following 312 nm UVB irradiation, are involved in the regulation of Treg function and differentiation and exert an anti-atherogenic effect (17, 22, 23). G protein-coupled receptor 32 and formyl-peptide receptor 2 were reported to mediate the favorable effect of these proresolving lipid mediators on Treg responses (22, 24). The augmented production of these lipid mediators in the skin may be a possible mechanism for UVB-dependent Treg expansion.

This study has several limitations. We observed that 312 nm UVB irradiation significantly attenuated atherosclerotic plaque formation and aortic inflammation in the aortic root but not in the thoracoabdominal aorta. The possible explanation for these discrepant results may be a difference in the atherosclerotic lesions analyzed or insufficient efficacy of this UVB treatment. Our 312 nm UVB wavelength resembles that of clinically used narrow-band UVB and therefore may be safely applied for the treatment of human atherosclerotic disease. However, effective irradiation conditions, including the area and dose for treating atherosclerotic disease, should be carefully examined before clinical use.

We demonstrated that clinically feasible 312 nm UVB irradiation effectively limits atherosclerotic plaque formation in the aortic root and improves the plaque stability by favorably modulating the T cell balance in lymphoid tissues and increasing the levels of proresolving lipid mediators in the skin. Our data may provide an attractive immunomodulatory approach for treating CAD.

## Figures and Tables

**Table I:** Antibodies for flow cytometry

Antibodies	Clone	Fluorescent dye	Source
anti-B220 Ab	RA3-6B2	PE	BD Biosciences 553090
anti-CD3 Ab	145-2C11	PECy7	BD Biosciences 552774
anti-CD3 Ab	500A2	V450	BD Biosciences 560801
anti-CD4 Ab	RM4-5	PECy7	BD Biosciences 552775
anti-CD8 Ab	53-6.7	PerCPCy5.5	BD Biosciences 553033
anti-CD11b Ab	M1/70	V450	BD Biosciences 560455
anti-CD11c Ab	HL3	V450	BD Biosciences 560521
anti-CD16/CD32 Ab	2.4G2	-	BD Biosciences 553142
anti-CD25 Ab	PC61	PE	BD Biosciences 553866
anti-CD44 Ab	IM7	PE	BD Biosciences 553134
anti-CD62L Ab	MEL-14	FITC	BD Biosciences 553150
anti-CD80 Ab	16-10A1	PE	BD Biosciences 553769
anti-CD86 Ab	GL1	APC	BD Biosciences 558703
anti-CD103 Ab	M290	FITC	BD Biosciences 557494
anti-CD152 Ab	UC10-4B9	APC	Thermo Fisher Scientific 17-1522-82
anti-Foxp3 Ab	FJK-16s	V450	Thermo Fisher Scientific 48-5773-82
anti-I-Ab Ab	AF6-120.1	FITC	BD Biosciences 553551
anti-IFN Ab	XMG1.2	PE	BD Biosciences 554412
anti-IL-10 Ab	JES5-16E3	APC	BD Biosciences 554468
anti-IL-17 Ab	TC11-18H10	APC	BD Biosciences 560184
anti-IL-4 Ab	11B11	PE	BD Biosciences 554435
anti-Ly6C Ab	AL-21	APC	BD Biosciences 560595
anti-Ly6G Ab	1A8	FITC	BD Biosciences 551460
anti-NK1.1 Ab	PK136	APC	BD Biosciences 550627

**Table II:** Body weight, plasma lipid profile, and plasma levels of CRP of 20-week-old 312 nm UVB-irradiated or nonirradiated male *Apoe*^−/−^ mice

	Nonirradiated	UVB 5 kJ/m^2^	UVB 10 kJ/m^2^
Body weight (g)	29.5 ± 2.9 (n = 11)	28.6 ± 2.6 (n = 11)	28.8 ± 2.5 (n = 11)
Total cholesterol (mg/dL)	600.2 ± 123.9 (n = 10)	678.5 ± 135.2 (n = 10)	701.8 ± 114.1 (n = 10)
LDL-cholesterol (mg/dL)	171.0 ± 34.1 (n = 10)	167.2 ± 39.1 (n = 10)	165.4 ± 46.7 (n = 10)
HDL-cholesterol (mg/dL)	17.7 ± 4.9 (n = 10)	20.2 ± 4.8 (n = 10)	19.2 ± 2.2 (n = 10)
Triglycerides (mg/dL)	85.5 ± 24 (n = 10)	89.7 ± 16 (n = 10)	118.1 ± 43.2 (n = 10)
CRP (μg/mL)	9.2 ± 1.2 (n = 10)	8.6 ± 0.5 (n = 10)	8.5 ± 0.9 (n = 10)

All the data are expressed as the mean ± s.d. *Apoe^−/−^* indicates apolipoprotein E-deficient; UVB, ultraviolet B; LDL, low-density lipoprotein; HDL, high-density lipoprotein; and CRP, C-reactive protein.

## References

[1] Soehnlein O, Libby P (2021). Targeting inflammation in atherosclerosis - from experimental insights to the clinic. Nat Rev Drug Discov.

[2] Ridker PM, Everett BM, Thuren T, MacFadyen JG, Chang WH, Ballantyne C (2017). Antiinflammatory Therapy with Canakinumab for Atherosclerotic Disease. N Engl J Med.

[3] Roy P, Orecchioni M, Ley K (2022). How the immune system shapes atherosclerosis: roles of innate and adaptive immunity. Nat Rev Immunol.

[4] Fernandez DM, Rahman AH, Fernandez NF, Chudnovskiy A, Amir ED, Amadori L (2019). Single-cell immune landscape of human atherosclerotic plaques. Nat Med.

[5] Buono C, Binder CJ, Stavrakis G, Witztum JL, Glimcher LH, Lichtman AH (2005). T-bet deficiency reduces atherosclerosis and alters plaque antigen-specific immune responses. Proc Natl Acad Sci U S A.

[6] Tanaka T, Sasaki N, Rikitake Y (2021). Recent Advances on the Role and Therapeutic Potential of Regulatory T Cells in Atherosclerosis. J Clin Med.

[7] Emoto T, Sasaki N, Yamashita T, Kasahara K, Yodoi K, Sasaki Y (2014). Regulatory/effector T-cell ratio is reduced in coronary artery disease. Circ J.

[8] Sasaki N, Yamashita T, Takeda M, Shinohara M, Nakajima K, Tawa H (2009). Oral anti-CD3 antibody treatment induces regulatory T cells and inhibits the development of atherosclerosis in mice. Circulation.

[9] Kita T, Yamashita T, Sasaki N, Kasahara K, Sasaki Y, Yodoi K (2014). Regression of atherosclerosis with anti-CD3 antibody via augmenting a regulatory T-cell response in mice. Cardiovasc Res.

[10] Kasahara K, Sasaki N, Yamashita T, Kita T, Yodoi K, Sasaki Y (2014). CD3 antibody and IL-2 complex combination therapy inhibits atherosclerosis by augmenting a regulatory immune response. J Am Heart Assoc.

[11] Takeda M, Yamashita T, Sasaki N, Nakajima K, Kita T, Shinohara M (2010). Oral administration of an active form of vitamin D3 (calcitriol) decreases atherosclerosis in mice by inducing regulatory T cells and immature dendritic cells with tolerogenic functions. Arterioscler Thromb Vasc Biol.

[12] Nilsson J, Hansson GK (2020). Vaccination Strategies and Immune Modulation of Atherosclerosis. Circ Res.

[13] Morita A (2018). Current developments in phototherapy for psoriasis. J Dermatol.

[14] Sasaki N, Yamashita T, Kasahara K, Fukunaga A, Yamaguchi T, Emoto T (2017). UVB Exposure Prevents Atherosclerosis by Regulating Immunoinflammatory Responses. Arterioscler Thromb Vasc Biol.

[15] Tanaka T, Sasaki N, Krisnanda A, Shinohara M, Amin HZ, Horibe S (2024). Novel UV-B Phototherapy With a Light-Emitting Diode Device Prevents Atherosclerosis by Augmenting Regulatory T-Cell Responses in Mice. J Am Heart Assoc.

[16] Tsuda S, Shinohara M, Oshita T, Nagao M, Tanaka N, Mori T (2017). Novel mechanism of regulation of the 5-lipoxygenase/leukotriene B(4) pathway by high-density lipoprotein in macrophages. Sci Rep.

[17] Fredman G, Hellmann J, Proto JD, Kuriakose G, Colas RA, Dorweiler B (2016). An imbalance between specialized pro-resolving lipid mediators and pro-inflammatory leukotrienes promotes instability of atherosclerotic plaques. Nat Commun.

[18] Korn T, Reddy J, Gao W, Bettelli E, Awasthi A, Petersen TR, Wucherpfennig KW, Strom TB, Oukka M, Kuchroo VK (2007). Myelin-specific regulatory T cells accumulate in the CNS but fail to control autoimmune inflammation. Nat Med.

[19] Hart PH, Norval M, Byrne SN, Rhodes LE (2019). Exposure to Ultraviolet Radiation in the Modulation of Human Diseases. Annu Rev Pathol.

[20] Furuhashi T, Saito C, Torii K, Nishida E, Yamazaki S, Morita A (2013). Photo(chemo)therapy reduces circulating Th17 cells and restores circulating regulatory T cells in psoriasis. PLoS One.

[21] Armstrong AW, Voyles SV, Armstrong EJ, Fuller EN, Rutledge JC (2011). A tale of two plaques: convergent mechanisms of T-cell-mediated inflammation in psoriasis and atherosclerosis. Exp Dermatol.

[22] Chiurchiu V, Leuti A, Dalli J, Jacobsson A, Battistini L, Maccarrone M (2016). Proresolving lipid mediators resolvin D1, resolvin D2, and maresin 1 are critical in modulating T cell responses. Sci Transl Med.

[23] Viola JR, Lemnitzer P, Jansen Y, Csaba G, Winter C, Neideck C (2016). Resolving Lipid Mediators Maresin 1 and Resolvin D2 Prevent Atheroprogression in Mice. Circ Res.

[24] Luan H, Wang C, Sun J, Zhao L, Li L, Zhou B (2020). Resolvin D1 Protects Against Ischemia/Reperfusion-Induced Acute Kidney Injury by Increasing Treg Percentages via the ALX/FPR2 Pathway. Front Physiol.

